# Proportion of infiltrating IgG-binding immune cells predict for tumour hypoxia

**DOI:** 10.1054/bjoc.2000.1650

**Published:** 2001-03

**Authors:** D R Collingridge, S A Hill, D J Chaplin

**Affiliations:** PET Oncology Group, Imperial College School of Medicine, Hammersmith Hospital, MRC Cyclotron Building, Du Cane Road, London, W12 0NN, UK; Tumour Microcirculation Group, Gray Laboratory Cancer Research Trust, PO Box 100, Mount Vernon Hospital, Northwood, Middlesex, HA6 2JR, UK; Oxigene Inc, 321 Arsenal Street, Watertown, Boston, MA 02472, USA

**Keywords:** hypoxia, macrophage, angiogenesis, pO _2_ histograph, flow cytometry

## Abstract

Macrophages can account for up to 50% of tumour mass and secrete many angiogenic factors. Furthermore, tumour hypoxia is thought to play a major role in the activation of macrophages and the regulation of angiogenesis. In this paper, we demonstrate a strong correlation between hypoxia and the recruitment of immune cells binding to IgG in 8 experimental tumours. We provide evidence that IgG binding immune cells in 3 tumour lines are predominately composed of macrophages. Reduced oxygenation may act as a stimulus for recruitment of immune cells to the tumour mass, and the detection of either IgG-positive host cells or macrophages may offer an alternative method for monitoring tumour hypoxia. © 2001 Cancer Research Campaign http://www.bjcancer.com


*British Journal of Cancer* (2001) **84**(5), 626–630
© 2001 Cancer Research Campaign
doi: 10.1054/ bjoc.2000.1650, available online at http://www.idealibrary.com on IDEAL

http://www.bjcancer.com

### Proportion of infiltrating IgG-binding immune cells predict for tumour hypoxia

**DR Collingridge[1], SA Hill[2] and DJ Chaplin[3]**

[1]PET Oncology Group, MRC Cyclotron Building, Imperial College School of Medicine, Hammersmith Hospital, Du Cane Road, London, W12 0NN, UK; [2]Tumour Microcirculation Group, Gray Laboratory Cancer Research Trust, PO Box 100, Mount Vernon Hospital, Northwood, Middlesex, HA6 2JR, UK; [3]Oxigene Inc, 321 Arsenal Street, Watertown, Boston, MA 02472, USA

**Summary** Macrophages can account for up to 50% of tumour mass and secrete many angiogenic factors. Furthermore, tumour hypoxia is thought to play a major role in the activation of macrophages and the regulation of angiogenesis. In this paper, we demonstrate a strong correlation between hypoxia and the recruitment of immune cells binding to IgG in 8 experimental tumours. We provide evidence that IgG binding immune cells in 3 tumour lines are predominately composed of macrophages. Reduced oxygenation may act as a stimulus for recruitment of immune cells to the tumour mass, and the detection of either IgG-positive host cells or macrophages may offer an alternative method for monitoring tumour hypoxia. © 2001 Cancer Research Campaign http://www.bjcancer.com

**Keywords**: hypoxia; macrophage; angiogenesis; $pO_2$ histograph; flow cytometry

Solid tumours are not a mass of neoplastic cells in isolation; rather, they are a complex matrix of many cellular types that often outnumber the tumour cell population. Indeed, macrophages can account for up to 50% of the tumour mass in breast carcinoma (Kelly et al, 1988; O'Sullivan and Lewis, 1994; Leek et al, 1994). Macrophages are known to be involved in the angiogenic process, and can be stimulated in hypoxic, avascular regions of the tumour microenvironment to secrete a wide range of angiogenic factors, including vascular endothelial growth factor (VEGF), basic fibroblast factor (bFGF), tumour necrosis factor α (TNFα), endothelial growth factor (EGF), interferons, and others (Leek et al, 1997). Additionally, tumour hypoxia alone can also alter the effects of growth factors and cytokines, induce transcription factors and DNA repair enzymes, and has been associated with increased tumour aggressiveness (Brizel et al, 1996; Dachs and Stratford, 1996). This led us to speculate that hypoxia may be acting as a stimulus for macrophage infiltration to help elicit the angiogenic response, and therefore the percentage of host cells within the tumour mass may correlate with, and provide a surrogate marker for, tumour hypoxia.

Tumour hypoxia is a major cause of radioresistance, and can also influence the efficacy of chemotherapy (Gray et al, 1953; Teicher et al, 1981). Measurements of oxygen tension in human tumours using polarographic microelectrodes have demonstrated the existence of large hypoxic fractions (Höckel et al, 1993; Brizel et al, 1996; Nordsmark et al, 1996; Collingridge et al, 1999), and other studies have shown that human tumour $pO_2$ can be modified to improve therapeutic response (Saunders and Dische, 1996; Hoskin et al, 1997). The use of polarographic electrodes is restricted however by their invasive nature, and their limited accessibility to superficial tumours or sites exposed during surgery. Deep-seated tumours of the bowel and digestive tract, for example, are not usually available for measurement. Non-invasive approaches utilizing MRI and PET are currently in development, and it is possible that surrogate markers that strongly correlate with tumour hypoxia may open other avenues of non-invasive $pO_2$ measurement. A routine method of assessing tumour oxygenation prior to, and during, therapy would allow monitoring of treatment efficacy, modification of treatment schedules as necessary, and individualization of therapy according to the patients' own physiology.

In this paper, we demonstrate that the infiltration of IgG-binding immune cells into solid experimental tumours strongly correlate with tumour hypoxia, and appear to be predominately derived from a macrophage lineage.

### MATERIALS AND METHODS

### Animals and tumour models

8 murine tumour models were used in this investigation. The anaplastic sarcoma F (SaF), and the adenocarcinomas NT (CaNT), X (CaX), and WW (CaWW) were transplanted subcutaneously into the rear dorsum of female CBA/Gy f TO mice. The methylcholanthrene-induced fibrosarcoma R (FsaR), the spontaneous fibrosarcoma N (FSaN), and the radiation-induced fibrosarcoma, RIF-1, were grown subcutaneously in the rear dorsum of female C3H/Gy f BXC/2 mice, and the adenocarcinoma RD (CaRD) was grown subcutaneously in the rear dorsum of female WHT/Gy f C57 B1 mice. SaF, CaNT, CaX, CaWW, and CaRD tumours arose spontaneously in the Gray Laboratory mouse colonies and were isolated and characterized between 1957 and 1980. FsaR and FsaN tumours were derived from the murine FSA fibrosarcoma (Milas et al, 1975, 1984), and the RIF-1 tumour was originally characterized by Twentyman et al (1980). All tumours, excluding RIF-1, were serially maintained by injecting 0.05 ml of a crude cell suspension prepared by mechanical dissociation of an excised

*Received 26 July 2000*
*Revised 20 November 2000*
*Accepted 30 November 2000*

*Correspondence to:* DR Collingridge tumour from a donor animal. RIF-1 tumours were inoculated by injecting $5 \times 10^5$ cells from exponentially growing cell cultures suspended in 0.05 ml of PBS. Tumours were selected for treatment when they had reached 5–8 mm in diameter (100–300 mg). All animal experiments were performed in full compliance with government regulations and UKCCCR guidelines on animal welfare.

### Immune cell determination

2 fluorescein-labelled antibodies were used to assess the proportion of infiltrating immune cells in each tumour line by flow cytometry. Cell suspensions were labelled with a non-specific polyclonal antibody (goat anti-mouse IgG/FITC; Sigma-Aldrich Company Ltd, Dorset, UK), which binds to macrophages and other polymorphs, to determine a gross indication of the immune cell content. Cell suspensions from the SaF, CaNT, and FSaN tumours where also incubated with a monoclonal antibody (rat anti-mouse F4/80/FITC; Serotec Ltd, Kidlington, Oxford, UK) directed against the macrophage-specific F4/80 antigen (Hume and Gordon, 1983). Tumour cell suspensions were obtained by excising solid tumours from host mice. A minimum of 3 tumours were assayed for each tumour line. The excised material was minced and enzymatically digested for 30 minutes at 37˚C with either Collagenase (type II; 2 mg $ml^{-1}$; activity, >125 U $mg^{-1}$), or Dispase (20 mg $ml^{-1}$; activity, >6 U $mg^{-1}$) dissolved in Hanks medium (Sigma-Aldrich Company Ltd, Dorset, UK). Both methods of digestion also contained DNase (type I; 0.2 mg $ml^{-1}$; activity, 420 U $mg^{-1}$). Following digestion, Hanks medium containing 7% fetal bovine serum was added to neutralize the enzymes and the samples were passed through a 25G needle and a 35 μm filter to obtain a single cell suspension. The suspensions were centrifuged and resuspended in cold PBS and placed on ice. All samples where kept ice-cold to inhibit polymorph activity and prevent phagocytosis of reagents.

To label the samples, cell suspensions were fixed with ice-cold 70% ethanol, and then washed and resuspended in PBS at a concentration of $7.5 \times 10^5$ cells $ml^{-1}$. To 2 ml aliquots, 20 μl of neat IgG/FITC or 80 μl of F4/80/FITC (diluted 1:4 in Trizma buffer) was added. Samples were then incubated on ice for 5 minutes (IgG/FITC) or 30 minutes (F4/80/FITC). Following incubation, samples were washed and resuspended in PBS and passed through a 25G needle. Finally, RNase (type I; 1 mg $ml^{-1}$; activity, 50–100 U $mg^{-1}$) and propidium iodide (1 mg $ml^{-1}$) were added, and the samples analysed by flow cytometry (FACScan; Becton-Dickinson UK Ltd, Cowley, Oxford, UK) to determine the proportion of cells positively labelled with FITC.

Immune cell distributions were assessed for the SaF and FSaN tumours using Hoechst 33342 (4 mg $ml^{-1}$; Sigma-Aldrich Company Ltd, Dorset, UK). This dye penetrates tumour tissue producing a gradient of fluorescence (visible in the u.v. range) that identifies the position of cells in situ relative to any functional vasculature (Chaplin et al, 1985). Mice were given 0.1 ml intravenous injections of Hoechst 33342 20 minutes prior to tumour excision. Tumours were then labelled for immune cell content as described earlier. Using a Vantage Cell Sorter (Becton-Dickinson UK Ltd, Cowley, Oxford, UK) the distribution of Hoechst staining amongst the immune cells positively labelled for FITC was determined.

### Measurement of oxygen tension

Oxygen measurements were performed using a $pO_2$ histograph (Eppendorf KIMOC 6650, Hamburg, Germany). Full operational details for this machine are described elsewhere (Collingridge et al, 1997). Briefly, un-anaesthetized tumour-bearing mice were restrained in Perspex jigs exposing the rear dorsum. A reference Ag/AgCl electrode (Medicotest UK Ltd, St Ives, Cambridgeshire, UK) was attached to a shaved region on the back of each animal, and the jig was placed on a thermal barrier (Vetko, CO, USA) to maintain core temperature. Using a net step length of 0.6 mm, 40–70 $pO_2$ measurements were made from 6 tracks through each tumour. 5–35 tumours were measured for each experimental model. All oxygen measurements were post-calibrated to account for barometric pressure and tumour temperature. The median $pO_2$ and the percentage of $pO_2$ readings < 2.5 mmHg was calculated for each tumour and then averaged for each experimental model.

### RESULTS

Figure 1 shows the results from a representative sarcoma F tumour labelled with both FITC conjugated IgG (Figure 1A) and FITC conjugated F4/80 (Figure 1B). The graphs depict similar cell distributions; with the upper populations representing positively stained immune cells. The number of IgG-positive cells, as a proportion of the entire cell population, was approximately 40% in this tumour model. Table 1 shows the immune cell content of the other tumours. The IgG-positive component of these tumours ranged from approximately 20% to 50%. F4/80 labelling of the CaNT and the FSaN tumours indicated that the immune component was composed almost entirely of macrophages. Preliminary F4/80 labelling data from the SaF tumour (also shown in Figure 1) is broadly similar to the CaNT and FSaN tumours, indicating that a significant proportion of the infiltrating IgG-positive immune cells in the SaF were also macrophages.

Intravenous injections of Hoechst 33342 stained cells according to their position within the tumour. Figure 2 shows the distribution of Hoechst 33342 labelling of the SaF. Tumour cells, with a low affinity for IgG (Figure 2A), showed a Hoechst 33342 labelling profile (Figure 2C), which indicated a large number of cells with a strong incorporation of the dye, whilst progressively fewer proportions of cells with lesser degrees of incorporation, in other words, the data shows a gradient of staining extending from functional vasculature into less vascular regions. Additionally, the Hoechst 33342 profile (Figure 2D) for the IgG labelled immune cells (Figure 2B) was near identical to the tumour cell labelling, thus indicating the presence of immune cells distant from the

**Table 1** Percentage of immune cells in 8 experimental murine tumours

| Tumour | IgG positive (%) | *SEM | F4/80 positive (%) | *SEM |
|---|---|---|---|---|
| CaX | 19.9 | 1.6 | – | – |
| FsaR | 33.9 | 6.3 | – | – |
| CaNT | 36.1 | 2.6 | 34.2 | 2.0 |
| SaF | 39.8 | 2.2 | 27.5α | – |
| CaWW | 47.7 | 4.4 | – | – |
| CaRD | 50.5 | 3.4 | – | – |
| RIF-1 | 51.0 | 1.9 | – | – |
| FSaN | 51.0 | 2.1 | 51.4 | 5.6 |

Minimum of 3 tumours per determination, except α = average of 2 tumours.
*Errors are ± 1 SEM

© 2001 Cancer Research Campaign

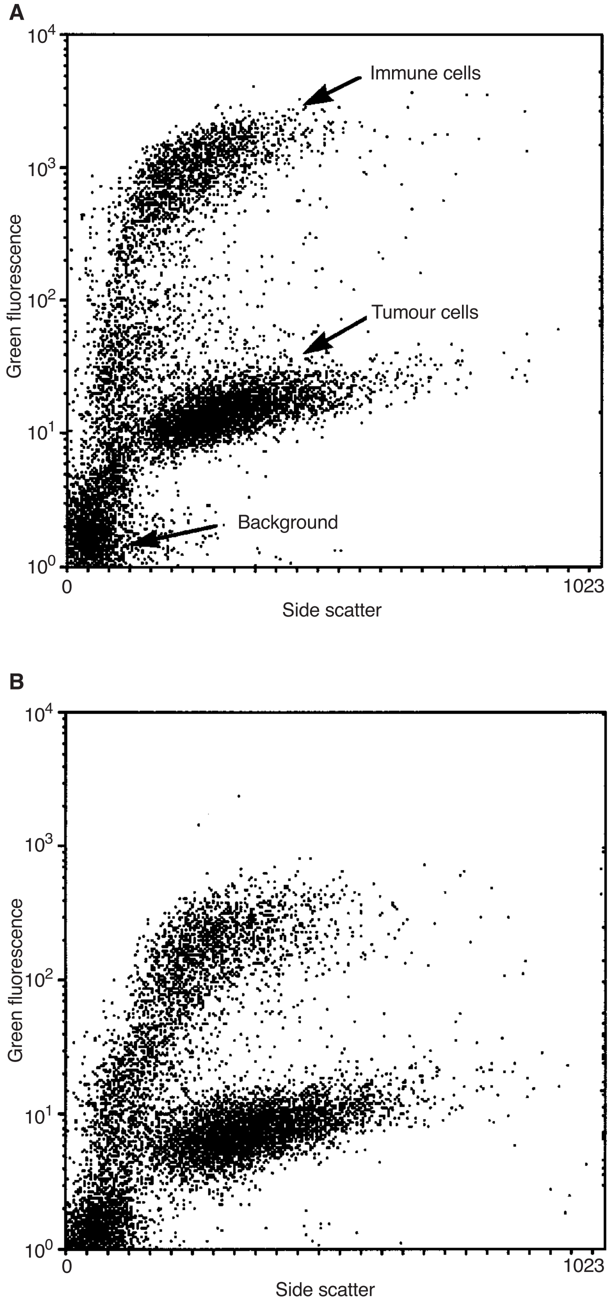

**Figure 1** Labelling of immune cells in a representative SaF tumour. Panel A: Sample labelled with polyclonal IgG antibodies. Panel B: Sample labelled with monoclonal antibodies directed against the macrophage-specific F4/80 antigen. Both antibodies were conjugated to FITC and cell labelling was assessed by flow cytometry

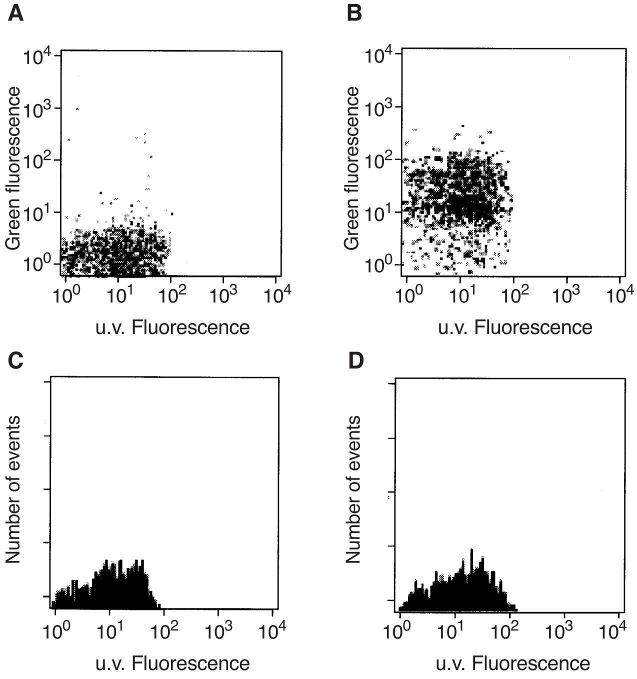

**Figure 2** Distribution of immune cells within a representative SaF tumour. Panel A: Tumour cells with low affinity to IgG-FITC. Panel B: Immune cells labelled with IgG-FITC. Panel C: Distribution of Hoechst 33342 vital dye amongst tumour cells. Panel D: Distribution of Hoechst 33342 vital dye amongst IgG-positive immune cells. Analysis was performed using flow cytometry vasculature. Experiments combining F4/80 with Hoechst 33342 to label macrophages within FSaN tumours showed similar results (data not shown).

Measurements of oxygen tension within the 8 types of tumour varied significantly. The median $pO_2$s ranged from 0.4 mmHg for the FSaN to 3.1 mmHg for the CaX, whilst the percentage of $pO_2$ readings less than 2.5 mmHg ranged from 46.3% to 89.4%. Figure 3 correlates the oxygen tensions of the solid tumours with their corresponding IgG-positive immune cell contents. There is a strong correlation between the 2 parameters indicating that with decreasing oxygenation, or conversely, increasing hypoxic fraction, the proportion of immune cells binding to IgG within the tumour mass increased.

### DISCUSSION

Macrophages represent a major component of the lymphoreticular infiltrate in tumours. In this paper, we demonstrate that up to 50% of the total tumour volume can be attributable to IgG-binding immune cells, which appear to be comprised predominately of macrophages. Other investigators have also reported large proportions of tumour-associated macrophages. Milas and colleagues (1987) have shown the macrophage content in a variety of murine tumours can vary from 9 to 83%, whilst Olive (1989) identified a macrophage content of between 20–60% in the murine SCCVII carcinoma. Similarly, tumour-associated macrophage content in human tumours can also be high. In a study of 20 human tumour biopsies of differing histology, Svennevig et al detected up to 28% of the biopsied tissue contained macrophages (1979). More recently, high proportions of macrophages have been measured in human breast carcinoma (Leek et al, 1997, 1999).

The data presented here does not allow us to comment on the absolute distribution of macrophages in the tumour mass. Hoechst 33342 stains cells relative to their distance from functional vasculature in accordance with the diffusion properties of the dye. The distribution profile shown in Figure 2D indicates that some cells were labelled weakly, and therefore they must have been at the limits of the diffusible range for Hoechst 33342. One can conclude that these cells must have migrated from the blood vessels into the

© 2001 Cancer Research Campaign

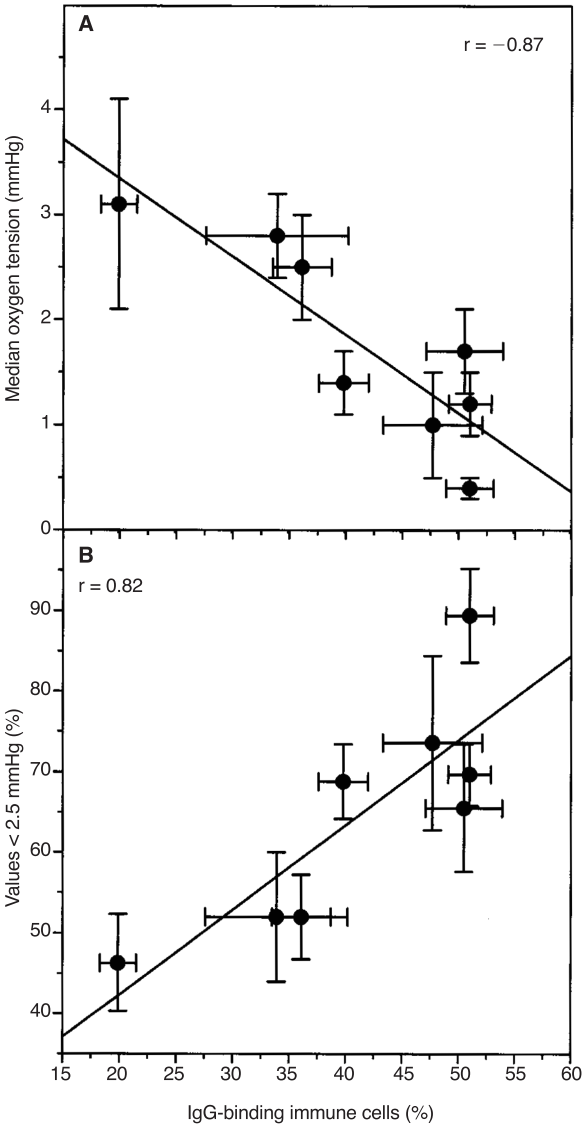

**Figure 3** Correlation between oxygen tension and the proportion of IgG-binding immune cells for eight murine tumours. Panel A: Average median $pO_2$ versus immune cell content. Panel B: Average percentage of $pO_2$ values <2.5 mmHg versus immune cell content. The fit is a linear regression, with the representative r value indicated in each panel. Errors are ± 1 S.E.M. $pO_2$ values derived from 5–35 tumours depending on cell line, immune content derived from a minimum of 3 tumours per cell line tumour mass; but the data does not provide any information regarding the proportion of cells in the avascular regions beyond the diffusible range of the dye, and it is therefore not possible to comment on the extent of infiltration of either IgG-positive immune cells or macrophage cells into the avascular necrotic regions of the tumour.

The role of macrophage recruitment to neoplastic tissue is complex, since macrophages have numerous functions related to tissue remodelling, inflammation, immunity and thrombosis. Thus they have the capacity to affect tumour vascularization, growth rate, stroma formation, and apoptotic cell death (Mantovani et al, 1992). Leek et al (1997) have shown that higher numbers of macrophages are found in angiogenic breast tumours and that they are concentrated in avascular, hypoxic regions of the tumour, consistent with many other reports indicating high macrophage numbers in avascular necrotic sites (Lewis et al, 1999). Hypoxia is known to stimulate the angiogenic activity of macrophages, and macrophage-derived VEGF is upregulated under low oxygen tensions (Harmey et al, 1998). Furthermore, the macrophage-derived peptide, PR39, can prevent degradation of hypoxia-inducible-factor 1$\alpha$ (HIF-1) and, because HIF-1 is an upstream regulator of VEGF, this can promote angiogenesis (Li et al, 1999; Ozaki et al, 2000).

It has been suggested, that macrophages are attracted to the tumour matrix by following a falling oxygen gradient or by chemoattractants released from stressed or necrotic cells (Leek et al, 1997). Consistent with these theories, breast cancer patients with low macrophage indices have a lower risk of recurrence and increased overall survival rates (Leek et al, 1997), furthermore, experimental studies have shown a trend between high macrophage numbers and radioresistance (Milas et al, 1987). These studies suggest that radiobiologically hypoxic tumours are targets for angiogenic activity, presumably to improve nutrient supply and drainage, and unsurprisingly have poor response rates to therapy. The results presented in this paper are in agreement with these conclusions. We show, amongst a range of experimental tumours, that those tumours exhibiting the highest levels of hypoxia also contain the highest proportions of IgG-binding immune cells. Furthermore, additional experimental data, that is not presented in this paper, provides us with little evidence to suggest that these cells are attracted to tumours containing high necrotic fractions to facilitate dead cell clearance; this leads us to conclude therefore, that hypoxia plays a key role in IgG-positive immune cell migration. It is important to highlight that the tumour models used in this study exhibit a range of oxygen tensions at the lower end of the spectrum, and further work is now needed to evaluate the immune cell content in well oxygenated tumours to examine the full dynamic range of the relationship. Furthermore, it would be useful to determine the strength of the relationship between tumours of the same type, and thereby examine a more clinically relevant endpoint, namely, whether one patient could be distinguished from another, rather than groups of patients from each other.

A strong correlation between IgG-positive immune cell content and tumour hypoxia may potentially offer an alternative method of measuring oxygenation. Non-invasive methods of detecting tumour hypoxia using MRI, EPR and PET are currently in development (Aboagye et al, 1998; O'Hara et al, 1998; Evans et al, 2000). PK11195, for example, binds to activated macrophages (Zovala and Lenfant, 1987), and PET studies using $^{11}$C-PK11195 at Hammersmith hospital are being conducted to image macrophage infiltration during neuro-inflammation in Rasmussen's encephalitis (Banati et al, 1999); thus it may be possible to utilize this imaging approach to indirectly measure tumour hypoxia in addition to macrophage activation states. Furthermore, macrophage measurement may evade the potential risks associated with many conventional nitroimidazole-based hypoxic markers, which can have mutagenic and carcinogenic effects (Voogd, 1981; Tocher, 1997).

In conclusion, this paper shows that the proportion of IgG-binding immune cells in solid tumours correlates with the degree of hypoxia, and that decreased oxygen gradients may act as a stimulus for cell recruitment to neoplastic tissue. Furthermore, a majority of the IgG-positive cells appear to be of a macrophage lineage, and thus the detection of macrophages may provide an indirect method of determining the hypoxic fraction in human tumours.

© 2001 Cancer Research Campaign 

### ACKNOWLEDGEMENTS

The Cancer Research Campaign (CRC) supported this work, and DRC would like to thank the Mount Vernon and Watford Hospitals Trust for financial support. The authors would also like to thank Dr George Wilson and his team for assisting with the FACS acquisitions, and Mr P Russell and his staff for care of the animals used in this study.

### REFERENCES

Aboagye EO, Kelson AB, Tracy M and Workman P (1998) Preclinical development and current status of the fluorinated 2-nitroimidazole hypoxia probe N-(2-hydroxy-3,3,3-trifluoropropyl)-2-(2-nitro-1-imidazolyl) acetamide (SR4554, CRC 94/17): a non-invasive diagnostic probe for the measurement of tumour hypoxia by magnetic resonance spectroscopy and imaging, and by positron emission tomography. *Anticanc Drug Des* **13**: 703–730

Banati RB, Goerres GW, Myers R, Gunn RN, Turkheimer FE, Kreutzberg GW, Brooks DJ, Jones T and Duncan JS (1999) [$^{11}$C](R)-PK11195 positron emission tomography imaging of activated microglia *in vivo* in Rasmussen's encephalitis. *Neurol* **53**: 2199–2203

Brizel DM, Scully SP, Harrelson JM, Layfield LJ, Bean JM, Prosnitz LR and Dewhirst DW (1996) Tumour oxygenation predicts for the likelihood of distant metastases in human soft tissue sarcoma. *Cancer Res* **56**: 941–943

Chaplin DJ, Durand RE and Olive PL (1985) Cell selection from a murine tumour using the fluorescent probe Hoechst 33342. *Br J Cancer* **51**: 569–572

Collingridge DR, Young WK, Vojnovic B, Wardman P, Lynch EM, Hill SA and Chaplin DJ (1997) Measurement of tumour oxygenation: a comparison between polarographic needle electrodes and a time-resolved luminescence-based optical sensor. *Radiat Res* **147**: 329–334

Collingridge DR, Piepmeier JM, Rockwell S and Knisely JPS (1999) Polarographic measurements of oxygen tension in human glioma and surrounding peritumoural brain tissue. *Radiother Oncol* **53**: 127–131

Dachs GU and Stratford IJ (1996) The molecular response of mammalian cells to hypoxia and the potential for exploitation in cancer therapy. *Br J Cancer* **74** (suppl. XXVIII): S126–S132

Evans SM, Kachur AV, Shiue CY, Hustinx R, Jenkins WT, Shive GG, Karp JS, Alavi A, Lord EM, Dolbier WR Jr. and Koch CJ (2000) Non-invasive detection of tumour hypoxia using the 2-nitroimidazole [$^{18}$F]EF1. *J Nucl Med* **41**: 327–336

Gray LH, Conger AD, Ebert M, Hornsey S and Scott OCA (1953) The concentration of oxygen dissolved in tissues at the time of irradiation as a factor in radiotherapy. *Br J Radiol* **26**: 638–648

Harmey JH, Dimitriadis E, Kay E, Redmond HP and Bouchier-Hayes D (1998) Regulation of macrophage production of vascular endothelial growth factor (VEGF) by hypoxia and transforming growth factor beta-1. *An Surg Oncol* **5**: 271–278

Höckel M, Knoop C, Schlenger K, Vorndran B, Baußmann E, Mitze M, Knapstein P-G and Vaupel P (1993) Intertumoural $pO_2$ predicts survival in advanced cancer of the uterine cervix. *Radiother Oncol* **26**: 45–50

Hoskin PJ, Saunders MI, Phillips H, Cladd H, Powell MEB, Goodchild K, Stratford MR and Rojas A (1997) Carbogen and nicotinamide in the treatment of bladder cancer with radical radiotherapy. *Br J Cancer* **76**: 260–263

Hume DA and Gordon S (1983) Mononuclear phagocyte system of the mouse defined by immunochemical localisation of antigen F4/80: identification of resident macrophages in medullary and cortical interstitium and the juxtaglomerular complex. *J Exp Med* **157**: 1704–1709

Kelly PM, Davison PS, Bliss E and McGee JO'D (1988) Macrophages in human breast tumours: a quantitative immunohistochemical study. *Br J Cancer* **57**: 174–177

Leek RD, Harris AL and Lewis CE (1994) Cytokine networks in solid tumours: regulation of angiogenesis. *J Leukoc Biol* **55**: 410–422

Leek RD, Lewis CE and Harris AL (1997) The role of macrophages in tumour angiogenesis. In: *Tumour Angiogenesis*, Bicknell R, Lewis CE and Ferrara N (eds). Oxford University Press

Leek RD, Landers RJ, Harris AL and Lewis CE (1999) Necrosis correlates with high vascular density and focal macrophage infiltration in invasive carcinoma of the breast. *Br J Cancer* **79**: 991–995

Lewis JS, Lee JA, Underwood JCE, Harris AL and Lewis CE (1999) Macrophage responses to hypoxia: relevance to disease mechanisms. *J Leuko Biol* **66**: 889–900

Li J, Post M, Volk R, Gao Y, Li M, Metais C, Sato K, Tsai J, Aird W, Rosenberg RD, Hampton TG, Li J, Sellke F, Carmeliet P and Simons M (2000) PR39, a peptide regulator of angiogenesis. *Nat Med* **6**: 49–55

Mantovani A, Bottazzi B, Collatta F, Sozzani S and Ruco L (1992) The origin and function of tumour-associated macrophages. *Immunol Today* **13**: 265–270

Milas L, Kogelnik HD, Basic I, Mason K, Hunter N and Withers HR (1975) Combination of *C. parvum* and specific immunization against artificial pulmonary metastases in mice. *Int J Cancer* **16**: 738–746

Milas L, Faykus MH Jr, McBride WH, Hunter N and Peters LJ (1984) Concomitant development of granulocytosis and enhancement of metastases formation in tumour-bearing mice. *Clin Exp Met* **2**: 181–190

Milas L, Wike J, Hunter N, Volpe J and Basic I (1987) Macrophage content of murine sarcomas and carcinomas: associations with tumour growth and tumour radiocurability. *Cancer Res* **47**: 1069–1075

Nordsmark M, Overgaard M and Overgaard J (1997) Pretreatment oxygenation predicts radiation response in advanced squamous cell carcinoma of the head and neck. *Radiother Oncol* **41**: 31–39

O'Hara JA, Goda F, Demidenko E and Swartz HM (1998) Effect on regrowth delay in a murine tumour of scheduling split-dose irradiation based on direct $pO_2$ measurements by electron paramagnetic resonance oximetry. *Radiat Res* **150**: 549–556

O'Sullivan and Lewis CE (1994) Tumour-associated leucocytes: friends or foes in breast carcinoma. *J Pathol* **172**: 229–235

Olive PL (1989) Distribution, oxygenation, and clonogenicity of macrophages in a murine tumour. *Cancer Commun* **1**: 93–100

Ozaki H, Yu AY, Della N, Ozaki K, Yamada H, Hackett SF, Okamoto N, Zack DJ, Semenza GL and Campochiaro PA (1999) Hypoxia inducible factor-1$\alpha$ is increased in ischaemic retina: temporal and spatial correlation with VEGF expression. *Invest Opthal Vis Sci* **40**: 182–189

Saunders MI and Dische S (1996) Clinical results of hypoxic cell radiosensitisation from hyperbaric oxygen to accelerated radiotherapy, carbogen and nicotinamide. *Br J Cancer* **74**(suppl XXVIII): S271–S278

Svennevig J-L, Lovik M and Woerner D (1979) Isolation and characterisation of lymphocytes and macrophages from solid, malignant human tumours. *Int J Cancer* **23**: 626–631

Teicher BA, Lazo JS and Sartorelli AC (1981) Classification of antineoplastic agents by their selective toxicities toward oxygenated and hypoxic tumour cells. *Cancer Res* **41**: 73–81

Tocher JH (1997) Reductive activation of nitroheterocyclic compounds. *Gen Pharmac* **28**: 485–487

Twentyman PR, Brown MJ, Gray JW, Franko AJ, Scoles MA and Kallman RF (1980) A new mouse tumour model system (RIF-1) for comparison of end-point studies. *J Natl Cancer Inst* **64**: 595–604

Voogd CE (1981) On the mutagenicity of nitroimidazoles. *Mutat Res* **86**: 243–277

Zavala F and Lenfant M (1987) Benzodiazepines and PK11195 exert immunomodulating activities by binding on a specific receptor on macrophages. *An New York Acad Sci* **496**: 240–249

 © 2001 Cancer Research Campaign
